# Optimization of Coriander Seed Oil Oleogel and Evaluation of Its Technological Performance in Dark Chocolate Formulation

**DOI:** 10.3390/foods15173134

**Published:** 2026-09-03

**Authors:** Zahra Nazari, Farzaneh Sabbagh

**Affiliations:** 1 Food Quality and Safety Research Department, Food Science and Technology Research Institute, ACECR—Razavi Khorasan Province, Mashhad 9177949367, Iran; 2 Department of Biosystems and Soft Matter, Institute of Fundamental Technological Research, Polish Academy of Sciences, 02-106 Warsaw, Poland

**Keywords:** oleogel, structured lipid system, *Coriandrum sativum* L., cocoa butter alternative, dark chocolate, D-optimal mixture design

## Abstract

Due to the high saturated fat content, increasing cost, and supply instability of cocoa butter (CB), the development of structured lipid systems with improved nutritional profiles and suitable technological functionality has received increasing attention. This study aimed to optimize a coriander seed oil-based oleogel (COG) using a D-optimal mixture design and evaluate its potential as a structured lipid phase for dark chocolate formulation. The effects of coriander seed oil, monoglycerides, candelilla wax, xanthan gum, and polyglycerol polyricinoleate (PGPR) on the physicochemical, rheological, and mechanical properties of the oleogel were investigated. The optimized COG formulation exhibited a maximum crystallization temperature of 30.159 °C, enhanced viscoelastic characteristics, and a stable three-dimensional lipid network. The optimized oleogel retained substantial amounts of bioactive compounds, including α-tocopherol, phenolic compounds, flavonoids, and linalool, together with antioxidant activity, and showed a lower accumulation of primary oxidation products during storage than the non-structured oil. Dark chocolate prepared with the optimized oleogel (COG-Ch) was evaluated in comparison with cocoa butter-based chocolate (CB-Ch) in terms of thermal behavior, rheological properties, texture, and storage-related whitening changes. Although COG-Ch showed comparable onset and maximum melting temperatures and a lower increase in whiteness index during storage at 35 °C, significant differences remained in melting enthalpy, hardness, and several rheological parameters compared with CB-Ch. These differences reflect the distinct lipid organization and crystallization behavior of the oleogel system. Therefore, the developed coriander seed oil oleogel should be considered a promising structured lipid phase for chocolate applications rather than a complete functional equivalent of cocoa butter. Further investigations involving sensory evaluation, crystal polymorphism analysis, microstructural characterization, and comprehensive oxidative stability assessment are required to determine its broader applicability in chocolate manufacturing.

## 1. Introduction

Chocolate is a complex multiphase food system in which the continuous fat phase plays a central role in determining texture, melting behavior, gloss, snap, and storage stability. Cocoa butter (CB) possesses a unique triacylglycerol (TAG) composition, primarily consisting of 1,3-dipalmitoyl-2-oleoylglycerol (POP), 1-palmitoyl-2-oleoyl-3-stearoylglycerol (POS), and 1,3-distearoyl-2-oleoylglycerol (SOS), which contributes to its characteristic crystallization behavior and the formation of desirable β-polymorphic crystals. These structural features are responsible for the rapid melting near body temperature, brittle texture, and glossy appearance associated with high-quality chocolate products [1]. However, cocoa butter functionality originates from a complex combination of TAG composition, solid fat content, polymorphism, and crystallization kinetics, making its complete replication challenging [2].

In addition to technological considerations, the availability and nutritional characteristics of cocoa butter have encouraged research into alternative lipid systems. Cocoa production is affected by geographical, climatic, and seasonal variations, which may contribute to fluctuations in cocoa butter supply and market price [2]. Furthermore, cocoa butter contains a relatively high proportion of saturated fatty acids, particularly palmitic and stearic acids, which has stimulated interest in developing structured lipid systems with improved fatty acid profiles [3]. Therefore, alternative fat systems are being investigated not only to reduce dependence on cocoa butter but also to introduce nutritionally favorable lipid sources into confectionery products.

Among plant-derived oils, coriander seed oil (*Coriandrum sativum* L.) represents an interesting candidate due to its distinctive fatty acid composition, particularly its high content of petroselinic acid, an uncommon positional isomer of oleic acid, as well as its naturally occurring bioactive compounds, including tocopherols and linalool. These compounds may contribute to improved nutritional characteristics and antioxidant potential [4]. However, the high degree of unsaturation of coriander seed oil limits its direct application in chocolate because liquid oils generally lack the solid fat content, crystallization behavior, and mechanical properties required for confectionery applications [5].

Oleogelation has emerged as a promising physical structuring approach to convert liquid oils into semi-solid lipid systems without hydrogenation or trans-fat formation. During oleogel formation, low concentrations of structuring agents such as waxes, monoacylglycerols, and polymers generate a three-dimensional network capable of immobilizing liquid oil [6]. This approach allows for the development of lipid materials with improved mechanical stability while maintaining the nutritional advantages of unsaturated oils [6].

Nevertheless, producing an oleogel that fully reproduces cocoa butter functionality remains a considerable challenge. Chocolate quality is governed by multiple interacting factors, including TAG composition, solid fat content, crystal polymorphism, rheological behavior, mechanical properties, and sensory perception. Therefore, similarity in a single physicochemical parameter, such as melting temperature, cannot be considered sufficient evidence of cocoa butter equivalence [7]. Accordingly, oleogel systems should be evaluated as structured lipid phases designed to provide selected technological functions required for chocolate processing rather than as direct replacements of cocoa butter.

The application of experimental design approaches, particularly mixture designs, provides an effective strategy for optimizing multi-component oleogel systems. D-optimal mixture designs enable the evaluation of interactions among formulation components while reducing the number of required experiments. However, the predictive capability of such models requires careful assessment through statistical evaluation and experimental validation within the investigated formulation space.

Therefore, the objectives of this study were to (i) optimize a coriander seed oil-based oleogel using a D-optimal mixture design by evaluating the effects of structuring components on physicochemical, rheological, and mechanical properties, and (ii) assess the technological performance of the optimized oleogel when incorporated into dark chocolate compared with conventional cocoa butter-based chocolate. The aim was not to establish complete functional equivalence with cocoa butter, but rather to determine whether coriander seed oil oleogel could provide a promising structured lipid phase with improved nutritional characteristics and suitable technological performance for chocolate applications.

## 2. Materials and Methods

### 2.1. Materials

Cold-pressed coriander seed oil (CSO) was obtained from Giahkala Company (Sabzevar, Iran). Cocoa butter (CB) was purchased from Altinmarka (Istanbul, Turkey) and used as the reference fat phase. Polyglycerol polyricinoleate (PGPR) was obtained from Palsgaard (Zierikzee, The Netherlands). Candelilla wax (Type A; melting point 68–72 °C), monoglycerides (purity > 95%), xanthan gum, and soy lecithin were supplied by Shimistore (Tehran, Iran). Analytical-grade solvents were purchased from Merck (Darmstadt, Germany). Cocoa powder and powdered sugar were obtained from local suppliers in Mashhad, Iran.

### 2.2. Experimental Design and Preparation of Coriander Seed Oil Oleogel (COG)

The formulation of coriander seed oil oleogel was optimized using a D-optimal mixture design generated by Design-Expert software (Version 13, Stat-Ease Inc., Minneapolis, MN, USA). This experimental approach was selected because the proportions of oleogel components were constrained to a fixed total composition (100%), and it allows efficient estimation of component effects and interactions within a limited number of experimental runs. Four formulation components were treated as independent mixture variables: coriander seed oil (CSO), monoglycerides (MG), candelilla wax (CW), and xanthan gum (XG). PGPR was maintained at a constant level of 2.00% in all formulations. The experimental ranges of the four variable components were CSO: 82–99%, MG: 0–10%, CW: 1–5%, and XG: 0–1%, with PGPR fixed at 2.00%. Thus, the total formulation, including the fixed PGPR content, was maintained at 100%. A total of 14 experimental formulations were generated by the D-optimal mixture design. Oleogel samples were prepared by dispersing the structuring agents into coriander seed oil, followed by heating at 65 °C with continuous stirring (1200 rpm) until complete melting and homogeneous dispersion were achieved. The samples were subsequently cooled under static conditions and stored at 4 °C for 24 h to allow network formation [8]. Following numerical optimization, the predicted optimum formulation was independently prepared in triplicate under identical processing conditions. The experimental responses were compared with model-predicted values to evaluate model performance and predictive accuracy.

### 2.3. Preparation of Dark Chocolate Containing Optimized Oleogel

Dark chocolates were prepared using either cocoa butter (CB-Ch) or optimized coriander seed oil oleogel (COG-Ch) as the lipid phase. Both formulations contained 50% powdered sugar, 35% lipid phase, 14.5% cocoa powder, and 0.5% soy lecithin.

For CB-Ch, cocoa butter was used as the sole fat phase, whereas COG-Ch was prepared by replacing the cocoa butter phase with the optimized oleogel. All ingredients except lecithin were mixed at 60 °C for 20 min under continuous stirring (200 rpm). Lecithin was then added, and mixing was continued under the same conditions.

For standardized thermal processing, both chocolate formulations were subjected to the same temperature sequence. The chocolate mass was cooled from 60 to 27 °C at a rate of 1.5 °C min^−1^ and subsequently reheated to 29 °C. This thermal treatment was applied to provide a consistent thermal history and reproducible processing conditions for comparison of CB-Ch and COG-Ch, rather than to assume identical crystallization mechanisms or polymorphic behavior in the two lipid systems. Since crystal polymorphism was not directly determined by X-ray diffraction, no conclusions regarding specific polymorphic forms were drawn.

The chocolate samples were molded at 29 °C and stored at 10 °C for 12 h before being wrapped in aluminum foil and stored under controlled conditions until analysis [9].

### 2.4. Determination of Bioactive Compounds

#### 2.4.1. Total Phenolic Content (TPC)

Total phenolic content was determined using the Folin–Ciocalteu method. Briefly, 0.5 mL of sample extract (10 mg/mL) was mixed with 2.5 mL of Folin–Ciocalteu reagent (0.2 N). After 5 min, 2 mL of sodium carbonate solution (75 g/L) was added. The mixture was incubated for 2 h, and absorbance was measured at 760 nm using a UV–Vis spectrophotometer (Agilent Technologies, Cary 60, Santa Clara, CA, USA). Gallic acid was used as the calibration standard, and results were expressed as mg gallic acid equivalents per gram of sample [10].

#### 2.4.2. Total Flavonoid Content (TFC)

Total flavonoid content was determined using the aluminum chloride colorimetric method. Sample extract (0.5 mL) was mixed with methanol, aluminum chloride solution (10%), potassium acetate (1 M), and distilled water. After incubation for 30 min at room temperature, absorbance was measured at 415 nm. Quercetin was used as the calibration standard [10].

#### 2.4.3. Linalool Determination by GC–MS

Linalool content was analyzed using gas chromatography–mass spectrometry (GC–MS, Agilent, Waldbronn, Germany). Samples were analyzed using an Agilent GC–MS system equipped with an HP-5MS capillary column (30 m × 0.25 mm × 0.25 μm). Helium was used as the carrier gas at 1 mL/min. The oven temperature program consisted of an initial temperature of 50 °C for 3 min, followed by heating to 200 °C at 5 °C/min and subsequently to 250 °C at 10 °C/min. Identification was performed by comparison of retention times and mass spectra with authentic standards and NIST library data [10].

#### 2.4.4. α-Tocopherol Determination by HPLC-DAD

α-Tocopherol was quantified using high-performance liquid chromatography equipped with a diode-array detector (HPLC-DAD, Acme 9000, Young Lin Instrument Co., Anyang, Republic of Korea). Separation was performed using a hexane/isopropanol mobile phase (99.5:0.5, *v*/*v*) at a flow rate of 1 mL/min. Detection was performed at 295 nm using tocopherol standards for calibration [10].

#### 2.4.5. Antioxidant Activity (DPPH Assay)

The antioxidant activity was evaluated using the DPPH radical scavenging method. Sample extracts at different concentrations were mixed with DPPH solution and incubated in darkness for 30 min. Absorbance was measured at 517 nm using a UV–vis spectrophotometer (Agilent Technologies, Cary 60, Santa Clara, CA, USA). The IC50 value was calculated as the concentration required to inhibit 50% of DPPH radicals [11].

### 2.5. Fatty Acid Composition

Fatty acid composition was determined by Agilent 7890A gas chromatograph (Agilent Technologies, Santa Clara, CA, USA) equipped with a flame ionization detector (GC–FID). Samples were converted to fatty acid methyl esters (FAMEs) before analysis. FAMEs were separated using an HP-88 capillary column. Identification and quantification were performed by comparison with authentic fatty acid standards [12].

### 2.6. Triacylglycerol Composition

Triacylglycerol species were analyzed using an Agilent 7890A gas chromatograph equipped with a flame ionization detector (FID) and a BPX70 capillary column (100 m × 0.25 mm i.d. × 0.2 μm film thickness). Nitrogen was used as the carrier gas at a flow rate of 4.41 mL/min. The initial oven temperature was set to 50 °C and increased to 220 °C at 50 °C/min, then to 290 °C at 30 °C/min, and finally to 380 °C at 50 °C/min, where it was held for 3 min. The injection was performed in split mode with a split ratio of 20:1. The injector and detector temperatures were both maintained at 380 °C. Samples were dissolved in hexane at a concentration of 10 mg/mL, and 2 μL of each sample was injected into the column. Triacylglycerol species were identified by comparison of their retention times with those of authentic standards and quantified using calibration curves [13].

### 2.7. Rheological and Mechanical Properties

Rheological measurements were performed using an Anton Paar MCR 301 rheometer (Graz, Austria) equipped with plate–plate geometry (40 mm upper plate, 61 mm serrated lower plate, and 1.5 mm gap). Samples were melted at 50 °C for 60 min and equilibrated at 20 °C before analysis.

The linear viscoelastic region was determined by amplitude sweep tests at 1 Hz. Frequency sweep measurements were subsequently performed within the identified linear region. Flow behavior was evaluated at 40 °C using increasing and decreasing shear rate ramps according to International Confectionery Association guidelines.

Casson yield stress and plastic viscosity were obtained by fitting the experimental data to the Casson model. Thixotropic behavior was evaluated from the hysteresis area between upward and downward flow curves [14].

Texture analysis was conducted using an Instron Universal Testing Machine (Hounsfield Test Equipment, Redhill, Surrey, UK) equipped with a 5 kg load cell and a 1.6 mm flat blade probe. Samples were equilibrated at 20 °C for 2 h before testing and penetrated at a constant speed of 1.5 mm/s to a depth of 5 mm. The maximum force recorded during penetration was taken as the measure of hardness [15].

### 2.8. Thermal Properties

T_mc_ was defined as the maximum crystallization temperature, corresponding to the peak temperature of the crystallization exotherm during the cooling scan in DSC. Thermal transitions were evaluated using differential scanning calorimetry (DSC 200 F3 Maia, NETZSCH-Gerätebau GmbH, Selb, Germany). Samples were heated from 0 to 150 °C at 5 °C/min under nitrogen atmosphere [16].

Slip melting point (SMP) was determined using the open capillary tube method [17].

Solid fat content (SFC) was measured by pulsed nuclear magnetic resonance (pNMR) using a Bruker Minispec mq20 (Bruker, Karlsruhe, Germany) after controlled melting and cooling, at 0, 5, 10, 20, 30, 35, and 40 °C [18].

### 2.9. Oxidative Stability

Primary oxidation was evaluated by peroxide value (PV) determination according to AOAC Method 965.33 [19]. Since accelerated oxidation tests were not performed, the obtained values were considered indicators of short-term oxidative stability rather than complete shelf-life prediction.

### 2.10. Color Measurement and Whiteness Index

Color parameters (L*, a*, b*) were measured after 1, 30, and 60 days of storage at 4, 25, and 35 °C. Whiteness index was calculated as an indirect indicator of whitening changes associated with bloom development [20]. Because direct evaluation of fat bloom requires microscopic and crystallographic analysis, WI values were interpreted as storage-related optical changes rather than direct confirmation of bloom formation.

### 2.11. Statistical Analysis

Experimental data from the mixture design were analyzed using Design-Expert software (Version 13). Response surface models were fitted using Scheffé’s polynomial equations suitable for mixture experiments.

Model adequacy was evaluated using analysis of variance (ANOVA), coefficient of determination (R^2^), adjusted R^2^, predicted R^2^, lack-of-fit test, and adequate precision. A non-significant lack-of-fit (*p* > 0.05) and adequate precision values greater than 4 were considered indicators of acceptable model performance. Multi-response optimization was performed using the desirability function approach. The optimum formulation was selected based on the highest overall desirability value within the experimental domain. Validation experiments were performed by preparing the optimized formulation independently in triplicate and comparing experimental responses with model predictions. Differences between formulations were evaluated using one-way ANOVA followed by Duncan’s multiple range test at *p* < 0.05 using using IBM SPSS Statistics software (Version 22, IBM Corp., Armonk, NY, USA).

## 3. Results and Discussion

### 3.1. Optimization of Coriander Seed Oil Oleogel (COG)

#### 3.1.1. Model Fitting and Statistical Validation

The effects of coriander seed oil (CSO), monoglycerides (MG), candelilla wax (CW), xanthan gum (XG), and PGPR on the physicochemical and rheological responses of the oleogel were evaluated using a D-optimal mixture design. The fitted models were selected based on statistical significance, coefficient of determination, adjusted and predicted R^2^ values, lack-of-fit evaluation, and adequate precision.

The obtained models showed satisfactory agreement between experimental and predicted values within the investigated formulation space (Table 1 and Table S1). The significance of the regression terms indicated that the structural components influenced the development of the oleogel network through different mechanisms, including hydrogen bonding and interactions between lipid and non-lipid structuring agents. The lack-of-fit tests were not significant (*p* > 0.05), indicating that the selected models adequately described the experimental responses. However, the predictive capability of mixture models is inherently dependent on the range and distribution of experimental formulations. Therefore, the developed models should be considered valid within the investigated formulation space, and additional validation using independent batches and expanded experimental conditions would be required for broader industrial prediction.

**Figure 1:**
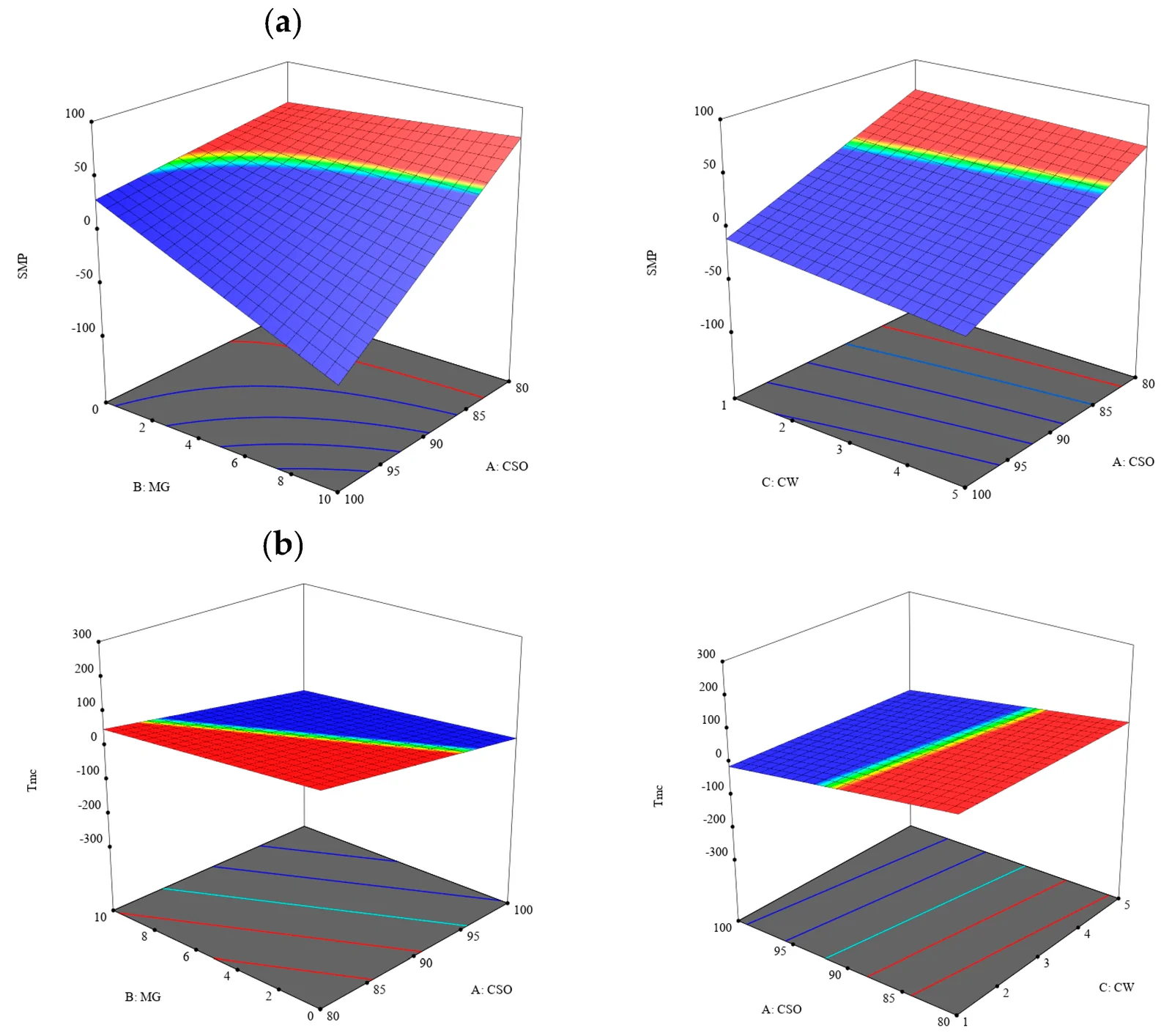

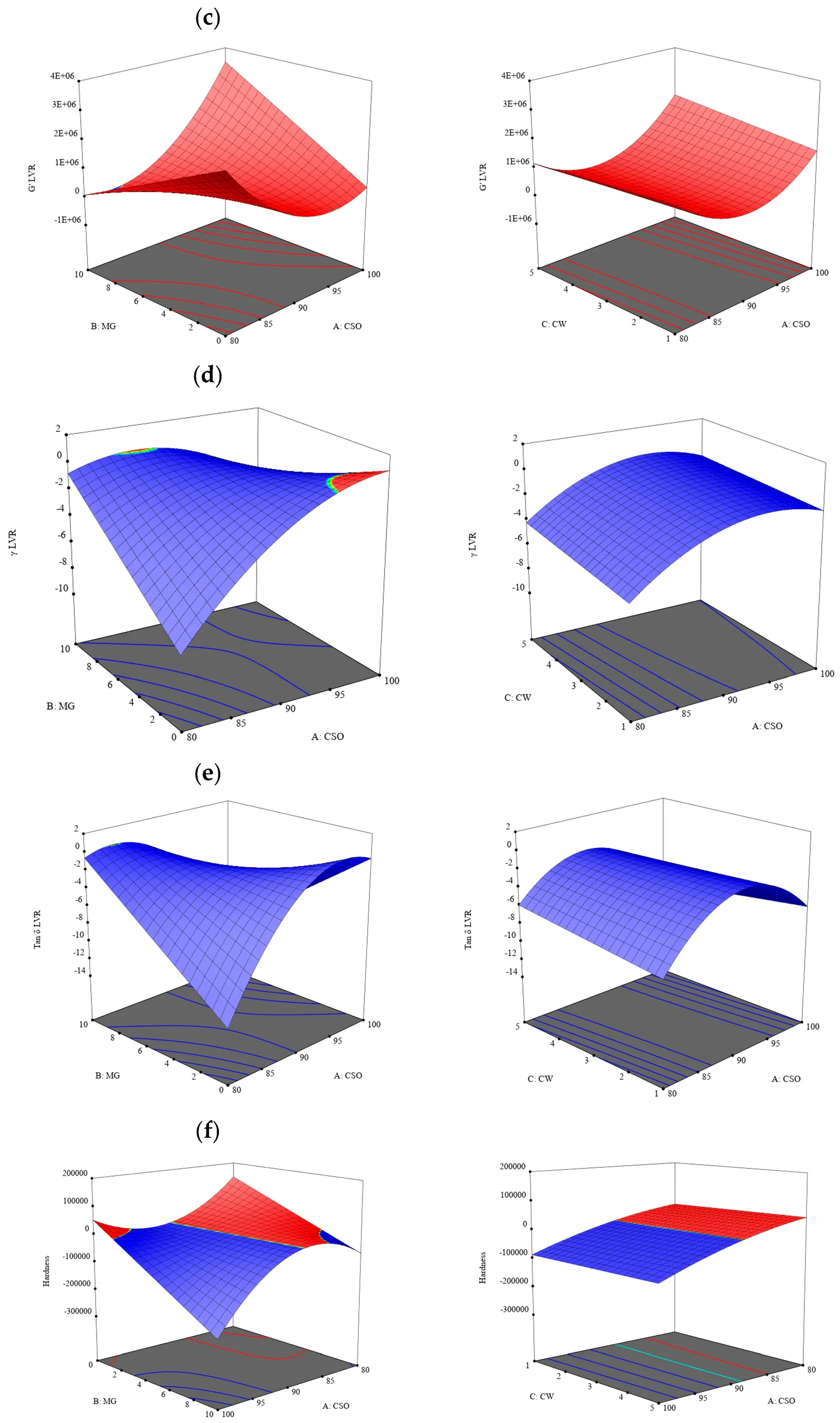
Surface responses representing the effect of coriander seed oil (CSO), monoglyceride (MG), and candelilla wax (CW) on SMP: Solid Melting Point (**a**); T_mc_: Maximum Crystallization Temperature (**b**); G′_LVR_: Storage Modulus at Linear Viscoelastic Region (**c**); γ _LVR_: Critical Strain at Linear Viscoelastic Region (**d**); tan δ _LVR_: Loss Tangent at Linear Viscoelastic Region (**e**); and hardness (**f**).

As shown in Figure 1a, the slip melting point (SMP) increased significantly with increasing candelilla wax content (p<0.05). Strong hydrogen bonding enhances the formation of stable polymeric crystalline networks and thermal resistance. Increasing monoglyceride concentration similarly elevated SMP by promoting denser crystal packing with their long saturated chains. PGPR enhanced SMP further by forming lipid-polymer complexes that improve molecular ordering [21]. DSC (T_mc_) confirmed SMP trends, showing increased melting temperatures in oleogels with higher candelilla wax and monoglyceride content, reflective of greater crystalline stability and reduced molecular disorder (Figure 1b). Although a slight increase was observed in the COG-based system, the difference was not statistically significant (p>0.05). PGPR further increased melting temperatures by facilitating a more ordered triglyceride network [22]. DSC analysis revealed melting temperatures comparable to cocoa butter. The storage modulus in the linear viscoelastic region (G′_LVR_) is an indicator of polymer network strength and oleogel functionality [23]. Candelilla wax significantly increased G′_LVR_ (p<0.05), likely due to hydrogen bond formation, while PGPR enhanced molecular organization and lipid-phase homogeneity (Figure 1c). γ _LVR_ also increased with PGPR and candelilla wax levels, showing improved resistance to deformation and extended elastic behavior (Figure 1d). Tan δ _LVR_, representing the viscous–elastic balance, decreased with increasing candelilla wax concentration, indicating dominance of elasticity due to well-structured hydrogen-bonded networks (Figure 1e). PGPR further reduced tan δ _LVR_ by promoting lipid-emulsifier mesomorphic phases with controlled molecular mobility. tan δ _LVR_ values of 0.01–0.03 have previously been associated with structured lipid systems exhibiting desirable viscoelastic characteristics for confectionery applications.

#### 3.1.2. Multi-Response Optimization and Experimental Validation

**Figure 2:**
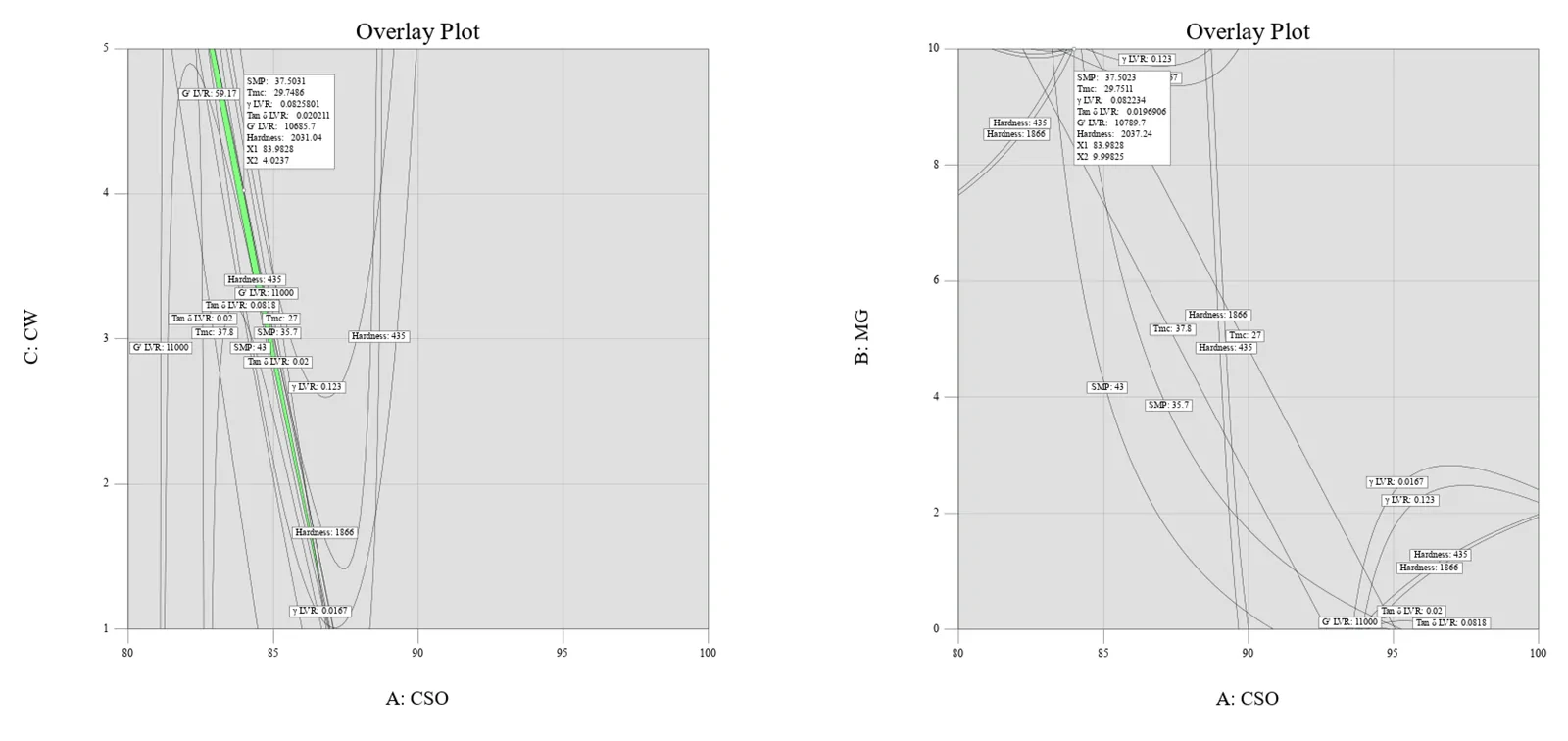
Formulation optimization plot showing the optimal region for achieving the desired responses. SMP: Solid Melting Point; T_mc_: Maximum Crystallization Temperature; G′_LVR_: Storage Modulus at Linear Viscoelastic Region; γ _LVR_: Critical Strain at Linear Viscoelastic Region; tan δ _LVR_: Loss Tangent at Linear Viscoelastic Region.

Multi-response optimization was performed using the desirability function approach to identify the formulation providing the most favorable combination of selected technological properties (Figure 2). The optimization criteria were established based on physicochemical and rheological parameters relevant to chocolate processing, including melting behavior, mechanical strength, and viscoelastic characteristics.

The desirability surface showed a relatively broad optimum region, indicating that multiple formulations could provide similar responses within the experimental design space. The selected formulation achieved an overall desirability value of 1.00, representing the optimum mathematical solution under the predefined constraints (Supplementary Table S2). Importantly, the desirability value reflects the ability of the model to satisfy the selected optimization criteria and does not indicate complete similarity or functional equivalence with cocoa butter. The optimized formulation contained 83.842% CSO, 9.996% MG, 3.996% CW, 0.159% XG, and 2.000% PGPR (Supplementary Table S3).

Experimental validation was conducted by independently preparing the optimized oleogel formulation. The predicted and experimental values showed generally good agreement for most responses, although a greater deviation was observed for γ _LVR_. Therefore, the validation supported the predictive performance of the models for most of the investigated responses within the experimental domain, while indicating lower predictive accuracy for γ _LVR_. Nevertheless, the observed agreement should be interpreted as validation of the statistical model rather than confirmation that the optimized oleogel reproduces the complete functionality of cocoa butter. The optimized COG exhibited technological characteristics approaching selected properties of cocoa butter, particularly in terms of melting behavior and viscoelastic structure. However, differences in solid fat content, melting enthalpy, rheological response, hardness, and crystallization-related properties demonstrate that the developed oleogel possesses a distinct structural organization compared with cocoa butter. Therefore, the optimized formulation should be regarded as a promising structured lipid system for chocolate applications rather than a direct replacement capable of fully reproducing cocoa butter functionality.

### 3.2. Characterization of Optimized Coriander Seed Oil Oleogel

#### 3.2.1. Thermal Behavior and Solid Fat Content

The optimized coriander seed oil oleogel exhibited a slip melting point (SMP) of 37.78 °C, which was comparable to that of cocoa butter (37.6 °C; *p* > 0.05). The optimized formulation also exhibited a predicted maximum crystallization temperature (T_mc_) of 30.159 °C, which was experimentally validated at 30.382 °C (Table 2). The similarity in SMP indicates that the oleogel undergoes a phase transition within a temperature range relevant for chocolate applications [24]. The observed thermal behavior can be attributed to the combined effects of monoglycerides and candelilla wax, which promote the formation of crystalline domains capable of immobilizing the liquid oil phase. Candelilla wax contributes to network formation through the development of crystalline structures, while monoglycerides may enhance lipid organization through intermolecular interactions [24].

However, melting temperature alone does not fully describe cocoa butter functionality. Chocolate performance depends on multiple thermal and structural parameters, including solid fat content, melting enthalpy, crystallization kinetics, and crystal polymorphism (Table 3).

Although the optimized oleogel showed a comparable SMP, its melting enthalpy was significantly lower than cocoa butter (approximately 42% reduction; *p* < 0.05). The lower ΔH_m_ may reflect differences in the amount and/or thermal organization of the crystalline phase in the oleogel compared with cocoa butter. This difference is consistent with the distinct structural mechanisms of the two lipid systems, in which cocoa butter contains a high proportion of crystallizable triacylglycerols, whereas the oleogel structure is generated primarily by a relatively low concentration of structuring agents within the liquid oil phase. Therefore, ΔH_m_ should not be interpreted as a direct measure of crystal content alone, but rather as an indicator of differences in the thermal characteristics of the crystalline phases. Solid fat content measurements further confirmed these structural differences. The optimized oleogel exhibited substantially lower SFC values compared with cocoa butter throughout the investigated temperature range. At 0 °C, COG showed approximately 22.5% solid fat compared with nearly 80% for cocoa butter. This difference reflects the distinct mechanisms responsible for structure formation: cocoa butter derives its firmness primarily from a high proportion of crystallizable saturated TAGs, whereas oleogels rely on a three-dimensional network formed by a relatively small amount of structuring agents. Therefore, the optimized oleogel demonstrated a suitable melting profile for chocolate-related applications but should not be considered thermally equivalent to cocoa butter.

#### 3.2.2. Rheological and Mechanical Properties

The rheological behavior of the optimized oleogel demonstrated the formation of a structured lipid network. The storage modulus (G′) remained higher than the loss modulus (G″) within the linear viscoelastic region, indicating predominantly elastic behavior characteristic of gel-like lipid structures (*p* < 0.05).

Increasing concentrations of candelilla wax enhanced the elastic response, which can be attributed to the formation of crystalline wax domains that reinforce the oleogel network. Monoglycerides also contributed to structural development through their ability to organize within the lipid phase and interact with other structuring components.

The optimized oleogel exhibited a significantly higher Casson yield stress than cocoa butter (*p* < 0.05), whereas no significant difference was observed in Casson plastic viscosity (*p* > 0.05). These findings suggest that the structured lipid network of the oleogel contributed to its higher resistance to flow initiation, as reflected by its increased yield stress, while its Casson plastic viscosity remained statistically comparable to that of cocoa butter. The increased network strength may enhance the oleogel’s resistance to deformation during handling; however, an excessively high yield stress could affect flow and processing behavior during chocolate manufacturing. Similarly, the hardness of COG was approximately 20% higher than that of cocoa butter (*p* < 0.05). The increased hardness can be associated with the reinforcing effect of candelilla wax crystals and the interconnected lipid network formed during oleogelation. This higher hardness indicates greater resistance to mechanical deformation; however, it should not necessarily be interpreted as a superior textural property, since the optimal hardness of an oleogel depends on its intended application.

Although these properties demonstrate successful conversion of coriander seed oil into a semi-solid structured lipid system, the differences in rheological and mechanical behavior highlight that the oleogel possesses a distinct physical organization compared with cocoa butter. Therefore, the optimized COG should be considered a promising structured fat phase with selected technological functionalities rather than a direct rheological replacement for cocoa butter.

#### 3.2.3. Bioactive Composition of Optimized Coriander Seed Oil Oleogel

The bioactive composition of coriander seed oil (CSO), cocoa butter (CB), and the optimized oleogel (COG) was evaluated to determine the retention of naturally occurring functional compounds after oleogel formation. Coriander seed oil exhibited higher concentrations of phenolic compounds, flavonoids, α-tocopherol, and linalool compared with cocoa butter, reflecting the characteristic phytochemical profile of this plant-derived oil (Table 4). Following oleogelation, the optimized COG retained considerable amounts of these bioactive compounds, although slight reductions were observed compared with the original coriander seed oil. These changes may be associated with thermal processing during oleogel preparation and interactions between bioactive compounds and the structured lipid matrix. The relatively high retention of TPC and TFC after oleogel preparation (approximately 87.5% and 91.0%, respectively) indicates that a substantial proportion of these bioactive compounds was retained following oleogelation. The antioxidant activity measured by the DPPH assay showed that CSO exhibited stronger radical scavenging capacity compared to cocoa butter, whereas the optimized oleogel maintained a considerable antioxidant potential. The preservation of antioxidant compounds in the oleogel may provide additional nutritional interest; however, DPPH radical scavenging activity represents chemical antioxidant capacity under experimental conditions and cannot be directly interpreted as evidence of enhanced product shelf-life or biological effects. Furthermore, the incorporation of the oleogel into chocolate involves additional thermal processing steps, including tempering and molding, which may influence the stability and availability of bioactive compounds. Therefore, future investigations should quantify the retention of individual bioactive compounds after chocolate processing and evaluate their contribution during storage. Overall, the results indicate that coriander seed oil provides a valuable source of naturally occurring bioactive compounds and that oleogelation allows partial preservation of these compounds within a structured lipid system. However, the nutritional advantages of the final chocolate product require further confirmation through comprehensive compositional and storage studies.

#### 3.2.4. Fatty Acid Composition and Triacylglycerol Profile

The fatty acid composition analysis demonstrated substantial differences between coriander seed oil and cocoa butter. Coriander seed oil contained a high proportion of unsaturated fatty acids, particularly petroselinic acid, oleic acid, and linoleic acid, resulting in a considerably lower saturated fatty acid content compared with cocoa butter (Table 4). The incorporation of monoglycerides and candelilla wax during oleogel formation increased the relative proportion of saturated fatty acids due to the presence of long-chain saturated components in the structuring agents. Nevertheless, the fatty acid profile of the optimized oleogel remained predominantly unsaturated, distinguishing it from the saturated TAG-rich composition of cocoa butter. Triacylglycerol analysis further confirmed the structural differences between the two lipid systems. Cocoa butter was characterized by the presence of major TAG species, including POP, POS, and SOS, which are responsible for its characteristic crystallization behavior and chocolate functionality. In contrast, these TAG species were absent or present at very low levels in coriander seed oil oleogel. The optimized oleogel contained TAG species derived mainly from the original coriander oil together with contributions from the added structuring agents. These compositional differences indicate that the crystallization mechanism and molecular organization of the oleogel differ fundamentally from cocoa butter. Therefore, although oleogelation improved the physical structure of coriander seed oil and enabled the development of a semi-solid lipid system, the resulting material should not be expected to reproduce the molecular characteristics of cocoa butter. The technological performance of the oleogel arises from the formation of a structured network rather than replication of cocoa butter TAG composition.

#### 3.2.5. Oxidative Stability of Optimized Oleogel

The oxidative stability of coriander seed oil and the optimized oleogel was evaluated by monitoring peroxide value (PV) during storage. Coriander seed oil showed an increase in PV from 0.91 to 2.81 mEq O_2_/kg after 60 days at 25 °C, indicating progressive lipid oxidation during storage (Table 5). In comparison, the optimized oleogel exhibited lower increases in peroxide value, with final values ranging from 0.91 to 1.61 mEq O_2_/kg under the same conditions (p<0.05). The lower accumulation of peroxide products in the optimized oleogel may be attributed to the immobilization of oil molecules within the oleogel network, which can reduce oxygen diffusion and limit molecular mobility. In addition, naturally occurring antioxidants in coriander seed oil, including tocopherols, may contribute to oxidative resistance. However, peroxide value represents only primary oxidation products and provides limited information regarding overall oxidative stability. Since accelerated oxidation analyses, such as the Rancimat or oxidative induction time measurements, were not performed, the present results should be interpreted as short-term oxidative behavior rather than as a prediction of shelf life. Further studies involving accelerated oxidation testing and long-term storage evaluation are necessary to determine whether the developed oleogel provides practical advantages during commercial chocolate storage.

### 3.3. Characterization of Dark Chocolate Containing Optimized Oleogel

#### 3.3.1. Thermal, Rheological, and Mechanical Properties of Oleogel-Based Chocolate

The thermal behavior of cocoa butter-based chocolate (CB-Ch) and coriander seed oil oleogel chocolate (COG-Ch) was evaluated to determine the influence of replacing the conventional fat phase with the structured lipid system. The slip melting point (SMP) of COG-Ch and CB-Ch was 38.8 ± 0.4 and 38.0 ± 0.4 °C, respectively, with no significant difference between the two formulations (*p* > 0.05) (Table 6). This comparable SMP indicates that both chocolate systems exhibited melting behavior within a temperature range relevant to chocolate applications. However, the melting enthalpy of COG-Ch was significantly lower than that of CB-Ch (13.9 ± 0.7 vs. 18.7 ± 1.0 J/g; *p* < 0.05). The lower enthalpy suggests differences in crystalline content and internal lipid organization between the two chocolate systems. Thus, although the two formulations showed comparable SMP values, the energy associated with their melting transitions was different, reflecting the distinct structural characteristics of the oleogel-based lipid phase. The hardness of COG-Ch was approximately 12% higher than that of CB-Ch (*p* < 0.05). This difference may result from the formation of a stronger structured lipid network generated by wax and monoglyceride crystals. While increased hardness may improve resistance to mechanical deformation, it may also affect fracture behavior and contribute to a firmer or potentially waxy oral texture at excessive levels. Because sensory evaluation was not conducted, the effect of these instrumental differences on consumer perception cannot be established. Therefore, texture measurements should be interpreted as indicators of mechanical performance rather than direct predictors of sensory quality.

The rheological behavior of COG-Ch also differed from that of CB-Ch, reflecting the different organization of the continuous fat phase. However, these rheological measurements were obtained at a single controlled temperature and therefore do not represent the temperature-dependent behavior of COG-Ch and CB-Ch across the 20–40 °C range. These findings demonstrate that the oleogel provides structural functionality in chocolate; however, it does not completely reproduce the rheological characteristics of cocoa butter.

#### 3.3.2. Whiteness Index and Storage-Related Whitening Changes

Changes in whiteness index (WI) were monitored during storage as an indirect indicator of surface whitening phenomena commonly associated with fat bloom development. Both CB-Ch and COG-Ch exhibited gradual changes in WI during storage at 4, 25, and 35 °C. The magnitude of WI increase differed between formulations, with COG-Ch showing a lower increase under certain storage conditions. The reduced increase in WI observed in the oleogel-based chocolate may be associated with the ability of the structured lipid network to limit oil migration and reduce lipid mobility during storage. However, WI measurements alone cannot confirm the formation, morphology, or mechanism of fat bloom development. Direct characterization of bloom formation requires complementary techniques, including polarized light microscopy, scanning electron microscopy, and X-ray diffraction analysis. Since these analyses were not performed in the present study, the observed WI changes should be interpreted as storage-related optical changes rather than definitive evidence of bloom inhibition. Overall, the incorporation of coriander seed oil oleogel resulted in chocolate with promising technological characteristics, including comparable melting temperature, acceptable mechanical stability, and modified storage-related whitening behavior. Nevertheless, differences in melting enthalpy, solid fat behavior, rheology, hardness, and molecular lipid composition confirm that the oleogel represents a distinct structured lipid system rather than a complete functional equivalent of cocoa butter. Future investigations combining sensory evaluation, crystallographic analysis, microstructural characterization, and industrial-scale processing trials are necessary to determine the practical applicability of oleogel-based chocolate formulations.

## 4. Conclusions

This study demonstrated the successful development and optimization of a coriander seed oil-based oleogel using a D-optimal mixture design approach. The incorporation of monoglycerides, candelilla wax, xanthan gum, and a fixed level of PGPR (2.0%) enabled the transformation of liquid coriander seed oil into a structured lipid system with enhanced mechanical strength and viscoelastic properties. The optimized oleogel also retained substantial amounts of coriander seed oil-derived bioactive compounds, including α-tocopherol, phenolic compounds, flavonoids, and linalool. Moreover, the lower increase in peroxide values during storage indicated improved oxidative stability of the structured oil compared with the non-structured coriander seed oil.

The optimized oleogel exhibited thermal and mechanical characteristics that supported its application as a structured lipid phase in chocolate. Chocolate formulated with the optimized oleogel showed a melting temperature comparable to that of cocoa butter-based chocolate and lower changes in whiteness under some storage conditions. However, measurable differences remained in important structural attributes, including solid fat content, melting enthalpy, hardness, rheological behavior, thixotropy, and triacylglycerol composition. These differences indicate that the developed oleogel does not fully reproduce the complex functionality of cocoa butter, which depends on its unique lipid composition, crystallization behavior, and microstructural organization.

The D-optimal mixture design provided an effective strategy for identifying a promising oleogel formulation within the investigated experimental range, and the close agreement between predicted and experimental values supported the adequacy of the developed models. The present data support the retention of bioactive compounds in the optimized oleogel; however, they do not establish the retention or bioactivity of these compounds in the final chocolate product.

Overall, coriander seed oil oleogel represents a promising structured lipid platform for chocolate formulation by providing a balance between technological functionality and improved fatty acid composition. Rather than being considered a complete cocoa butter substitute, this system may serve as an alternative structured fat phase for chocolate applications that can be further optimized for the development of healthier and more sustainable chocolate products. Further research involving independent validation, scale-up studies, sensory evaluation, crystal polymorphism analysis using X-ray diffraction, microstructural characterization, and long-term oxidative stability assessment is warranted to determine its practical applicability and consumer acceptance.

## Figures and Tables

**Table 1:** Mathematical models and statistical validation for the response variables.

Response	Model Equation	$R^{2}$	Adj-$R^{2}$	F-Value	*p*-Value
SMP	Y=+22.78−43.00A−21.47B−7.18C−2.18D−28.13AB−3.15AC−7.39BC−4.59BD	0.97	0.92	18.82	<0.006
$T_{mc}$	Y=+29.72−45.63A−23.91B−6.78C−2.41D	0.85	0.79	12.99	<0.001
${G^{\prime }}_{LVR}$	${log}_{10}Y=+5.61+5.44A+5.15B+4.77C+4.50D+6.15AB+5.39AC+5.18AD+5.21BC+4.98BD+6.089A^{2}$	0.98	0.92	16.85	<0.02
$\gamma_{LVR}$	$Y=-0.9073+1.71A+0.8368B-3.09AB-2.13A^{2}$	0.98	0.94	25.07	<0.01
${\tan\delta }_{LVR}$	$Y=-1.85-6.37AB-1.05AC-5.17A^{2}$	0.97	0.86	9.45	<0.04
Hardness	${log}_{10}Y=-3.69-4.83A-3.61B+1.55C-3.57D-4.32AB-3.61AC-2.79AD-3.42BC-3.34BD$	1	NA		

SMP: Solid Melting Point; T_mc_: Maximum Crystallization Temperature; G′_LVR_: Storage Modulus at Linear Viscoelastic Region; γ _LVR_: Critical Strain at Linear Viscoelastic Region; tan δ _LVR_: Loss Tangent at Linear Viscoelastic Region. A: CSO; B: MG; C: CW, and D: XG. PGPR was maintained at a constant level of 2.00% and was not included as an independent variable in the mixture model.

**Table 2:** Comparison between predicted and experimental values of physicochemical and rheological properties of the optimized formulation.

	SMP (°C)	T_mc_ (°C)	γ _LVR_ (%)	tan δ _LVR_	G′ _LVR_ (Pa)	Hardness (g)
Predicted	37.788 ^a^	30.159 ^a^	0.075 ^a^	0.022 ^a^	9998.845 ^a^	1877.260 ^a^
Experimental	37.741 ^a^	30.382 ^a^	0.024 ^a^	0.020 ^a^	9963.56 ^a^	1820.8 ^a^

SMP: Solid Melting Point; T_mc_: Maximum Crystallization Temperature; G′_LVR_: Storage Modulus at Linear Viscoelastic Region; γ _LVR_: Critical Strain at Linear Viscoelastic Region; tan δ _LVR_: Loss Tangent at Linear Viscoelastic Region. Values with the same superscript letter within a column are not significantly different (*p* > 0.05).

**Table 3:** Thermal, Rheological, and Mechanical Properties of CB and COG.

	CB	COG
Thermal behavior		
T_mo_ (°C)	19.6 ± 0.2 ^a^	17.7 ±0.2 ^b^
T_mp_ (°C)	27.7 ± 0.3 ^b^	29.8 ± 0.3 ^a^
SMP (°C)	37.6 ± 0.4 ^a^	37.78 ± 0.4 ^a^
ΔH_m_ (J/g)	64.8 ± 3.2 ^a^	37.0 ± 1.9 ^b^
Solid fat content (%)		
0 °C	80.25 ± 2.5 ^a^	22.5 ± 0.5 ^b^
5 °C	79.13 ± 3.2 ^a^	10.0 ± 0.3 ^b^
10 °C	75.15 ± 2.6 ^a^	8.55 ± 0.1 ^b^
20 °C	70.11 ± 2.2 ^a^	4.8 ± 0.01 ^b^
30 °C	24.6 ± 0.9 ^a^	3.18 ± 0.02 ^b^
35 °C	10.19 ± 0.001 ^a^	1.6 ± 0.001 ^b^
40 °C	0 ^a^	0 ^a^
Rheological and mechanical properties		
σ_Ca_ (Pa)	0.00004 ± 0 ^b^	0.99 ± 0.05 ^a^
η_Ca_ (Pa.s)	0.02 ± 0.002 ^a^	0.54 ± 0.03 ^a^
Thixotropy (Pa)	0.007 ± 0 ^b^	2.81 ± 0.2 ^a^
Hardness (g) (20 °C)s	1564 ± 51 ^b^	1877 ± 61 ^a^

Values are expressed as means of triplicate for each experimental replicate. ^a,b^ Means within each column with different superscripts are significantly different (*p* < 0.05). CB, cocoa butter; COG, coriander seed oil oleogel; T_mo_, onset melting temperature; T_mp_, peak melting temperature; and SMP, slip melting point; ΔH_m_, melting enthalpy; σ_Ca,_ Casson yield stress; and η_Ca,_ Casson plastic viscosity. Values with the same superscript letter within a column are not significantly different (*p* > 0.05).

**Table 4:** Nutritional Composition and Triacylglycerol Species of CSO, CB, and COG.

	CSO	CB	COG
Bioactive composition			
α-Tocopherol (mg/kg)	160.6 ± 1.14 ^a^	20.4 ± 0.82 ^c^	144.5 ± 0.31 ^b^
TPC (mg GAE/kg)	400.3 ± 2.21 ^a^	51.2 ± 1.81 ^c^	350.2 ± 0.13 ^b^
TFC (mg CE/kg)	220.1 ± 0.63 ^a^	28.3 ± 0.41 ^c^	200.2 ± 0.13 ^b^
Linalool (mg/kg)	6120 ± 50 ^a^	N.D. ^c^	5100 ± 40 ^b^
IC50 (DPPH, µg/mL)	40.2 ± 1.42 ^b^	315.3 ± 1.63 ^a^	44.5 ± 2.01 ^b^
Fatty Acid (g/100 g) composition			
C16:0 (palmitic)	3.9 ± 0.12 ^c^	27.1 ± 0.81 ^a^	7.2 ± 0.22 ^b^
C18:0 (stearic)	2.9 ± 0.09 ^c^	36.5 ± 1.10 ^a^	3.6 ± 0.12 ^b^
C18:1 (n9, oleic)	8.5 ± 0.26 ^b^	33.7 ± 1.01 ^a^	8.0 ± 0.24 ^b^
C18:1 (n12)	68.0 ± 2.04 ^a^	0.0 ± 0.00 ^c^	65.0 ± 1.95 ^b^
C18:2 (linoleic)	16.7 ± 0.50 ^a^	2.7 ± 0.08 ^b^	16.2 ± 0.48 ^a^
USFA (unsaturated)	93.2 ± 1.86 ^a^	36.4 ± 1.09 ^c^	89.2 ± 1.78 ^b^
SFA (saturated)	6.8 ± 0.20 ^c^	63.6 ± 1.91 ^a^	10.8 ± 0.32 ^b^
Triacylglycerol species			
Dipalmitoyl oleoyl glycerol (POP)	ND^b^	18.8 ± 0.5 ^a^	ND^b^
Palmitoyl oleoyl stearoyl glycerol (POS)	ND^b^	38.9 ± 0.8 ^a^	ND^b^
Distearoyl oleoyl glycerol (SOS)	ND^b^	27.8 ± 0.6 ^a^	ND^b^
Dioleoyl palmitoyl glycerol (OOP)	ND^b^	2.8 ± 0.1 ^a^	ND^b^
Stearoyl oleoyl oleoylglycerol (SOO)	ND^b^	3.8 ± 0.2 ^a^	ND^b^
Dipetroselinoyl palmitoyl glycerol (PPsPs)	2.9 ± 0.08 ^a^	ND^b^	2.5 ± 0.5 ^a^
Tripetroselinin (PsPsPs)	39.6± 0.5 ^a^	ND^b^	34± 0.2 ^a^
dipetroselinoyl oleoyl glycerol (OPsPs)	17.1± 0.3 ^a^	ND^b^	14.5± 0.3 ^a^
Dipetroselinoyl linoleoyl glycerol (PsPsL)	22.6± 0.3 ^a^	ND^b^	18.2± 0.2 ^a^

N.D.: Not detected. Values are expressed as means of triplicate for each experimental replicate. ^a–c^ Means within each column with different superscripts are significantly different (*p* < 0.05). CSO, coriander seed oil; CB, cocoa butter; COG, coriander seed oil oleogel. Values with the same superscript letter within a column are not significantly different (*p* > 0.05).

**Table 5:** Peroxide Values (mEq O_2_/kg) of Coriander Seed Oil and Oleogel during Storage.

Temperature	Day	CSO	COG
4 °C			
	1	0.91 ± 0.02 ^a^	0.91 ± 0.02 ^a^
	30	1.08 ± 0.03 ^a^	0.92 ± 0.03 ^b^
	60	1.91 ± 0.03 ^a^	0.98 ± 0.03 ^b^
25 °C			
	1	0.91 ± 0.03 ^a^	0.91 ± 0.03 ^a^
	30	2.07 ± 0.06 ^a^	1.30 ± 0.04 ^b^
	60	2.81 ± 0.07 ^a^	1.61 ± 0.05 ^b^

Values are expressed as means of triplicate for each experimental replicate. ^a,b^ Means within each column with different superscripts are significantly different (*p* < 0.05). CSO, coriander seed oil; COG, coriander seed oil oleogel. Values with the same superscript letter within a column are not significantly different (*p* > 0.05).

**Table 6:** Physical Properties of CB-Ch and COG-Ch.

		CB-Ch	COG-Ch
Thermal Behavior			
T_mo_ (°C)		19.6 ± 0.2 ^a^	19.5 ± 0.2 ^a^
T_mp_ (°C)		28.0 ± 0.3 ^a^	32.0 ± 0.3 ^a^
SMP (°C)		38.0 ± 0.4 ^a^	38.8 ± 0.4 ^a^
ΔH_m_ (J/g)		18.7 ± 1.0 ^a^	13.9 ± 0.7 ^b^
Rheological and Mechanical Properties			
σ_Ca_ (Pa)		4.35 ± 0.23 ^b^	7.04 ± 0.36 ^a^
η_Ca_ (Pa.s)		0.88 ± 0.04 ^a^	0.89 ± 0.04 ^a^
Thixotropy (Pa)		11.3 ± 0.3 ^b^	22.00 ± 1.2 ^a^
Hardness (g)		1203 ± 60 ^b^	1356 ± 0.8 ^a^
Whiteness Index			
4 °C	day		
	1	20.01 ± 0.20 ^a^	20.31 ± 0.20 ^a^
	30	20.29 ± 0.21 ^b^	21.55 ± 0.22 ^a^
	60	20.71 ± 0.21 ^a^	21.80 ± 0.22 ^a^
25 °C			
	1	20.01 ± 0.20 ^a^	20.31 ± 0.20 ^a^
	30	21.32 ± 0.22 ^a^	22.65 ± 0.23 ^a^
	60	23.62 ± 0.24 ^a^	24.08 ± 0.24 ^a^
35 °C			
	1	20.01 ± 0.20 ^a^	20.31 ± 0.20 ^a^
	30	25.90 ± 0.26 ^a^	21.99 ± 0.22 ^b^
	60	28.16 ± 0.28 ^a^	25.21 ± 0.25 ^b^

Values are expressed as the mean of triplicate measurements for each experimental replicate. ^a,b^ Means within each column with different superscripts are significantly different (*p* < 0.05). CB-Ch, cocoa butter-based chocolate; COG-Ch, Coriander seed oil oleogel chocolate; T_mo_, onset melting temperature; T_mp_, peak melting temperature; SMP, slip melting point; ΔH_m_, melting enthalpy; σ_Ca,_ Casson yield stress; and η_Ca,_ Casson plastic viscosity. Values with the same superscript letter within a column are not significantly different (*p* > 0.05).

## Data Availability

The data supporting the findings of this study are available from the corresponding authors upon reasonable request.

## Supplementary Materials

The following supporting information can be downloaded at: https://www.mdpi.com/article/10.3390/foods15173134/s1.
