# Supplementary material for: Optimization of Coriander Seed Oil Oleogel and Evaluation of Its Technological Performance in Dark Chocolate Formulation
Source: Foods. 2026 Sep 3;15(17):3134. doi: 10.3390/foods15173134 (PMC13565070; doi:10.3390/foods15173134)
Supplement: Supplementary file 1 [file foods-15-03134-s001.zip › foods-4492807-supplementary.pdf]

**Supplementary Table S1.**

| Run | Response 1 | Response 2 | Response 3 | Response 4 | Response 5 | Response 6 |
|---|---|---|---|---|---|---|
| | SMP | $T_{mc}$ | $\gamma_{LVR}$ | Tan $\delta_{LVR}$ | $G'_{LVR}$ | Hardness |
| 1 | 35.7 | 29.6 | 0.021 | 0.025 | 59.17 | 435 |
| 2 | 37 | 30.8 | 0.1149 | 0.05 | 1980 | 526 |
| *3* | *39* | *31.6* | *0.123* | *0.0818* | *5180* | *1773* |
| 4 | 38.5 | 31.3 | 0.0598 | 0.028 | 3160 | 656 |
| 5 | 41.5 | 32.4 | 0.063 | 0.028 | 3440 | 1345 |
| 6 | 42 | 37 | 0.0581 | 0.0328 | 7480 | 1598 |
| 7 | 43 | 37.8 | 0.0893 | 0.0355 | 4880 | 1526 |
| 8 | 36.4 | 27 | 0.0167 | 0.02 | 2518.83 | 586 |
| 9 | 37 | 28 | 0.0931 | 0.02 | 8340 | 1644 |
| 10 | 38.5 | 31.3 | 0.046 | 0.025 | 11000 | 1866 |
| 11 | 38.5 | 31.2 | 0.05 | 0.0368 | 1358 | 493 |
| 12 | 38.5 | 31 | 0.0527 | 0.0345 | 2480 | 662 |
| 13 | 40.7 | 32 | 0.0272 | 0.033 | 6480 | 686 |
| 14 | 41.4 | 34.6 | 0.0439 | 0.033 | 4320 | 1413 |

**Supplementary Table S2.**

Constraints

| Name | Goal | Lower Limit | Upper Limit | Lower Weight | Upper Weight | Importance |
|---|---|---|---|---|---|---|
| A:CSO | is in range | 80 | 100 | 1 | 1 | 3 |
| B:MG | is in range | 0 | 10 | 1 | 1 | 3 |
| C:CW | is in range | 1 | 5 | 1 | 1 | 3 |
| D:XG | is in range | 0 | 1 | 1 | 1 | 3 |
| E:PGPR | Fixed at | 2 | 2 | - | - | - |
| SMP | is in range | 36 | 38 | 1 | 1 | 3 |
| $T_{mc}$ | is in range | 27 | 37.8 | 1 | 1 | 3 |
| $\gamma_{LVR}$ | is in range | 0.0167 | 0.123 | 1 | 1 | 3 |
| Tan $\delta_{LVR}$ | is in range | 0.02 | 0.0818 | 1 | 1 | 3 |
| $G'_{LVR}$ | is in range | 59.17 | 11000 | 1 | 1 | 3 |
| Hardness | maximize | 435 | 1866 | 1 | 1 | 3 |

A = CSO, B = MG, C = CW, and D = XG. PGPR was maintained at a constant level of 2.00% and was not included as an independent variable in the mixture model.

**Supplementary Table S3.**

| Number | CSO | MG | CW | XG | PGPR | SMP | $T_{mc}$ | $\gamma_{LVR}$ | Tan $\delta_{LVR}$ | $G'_{LVR}$ | Hardness | Desirability | |
|---|---|---|---|---|---|---|---|---|---|---|---|---|---|
| 1 | 83.842 | 9.996 | 3.996 | 0.159 | 2.000 | 37.788 | 30.159 | 0.075 | 0.022 | 9998.845 | 1877.260 | 1.000 | Selected |
| 2 | 83.678 | 9.996 | 3.692 | 0.622 | 2.000 | 37.830 | 30.820 | 0.071 | 0.023 | 9566.264 | 1961.643 | 1.000 | |
| 3 | 83.948 | 9.998 | 4.043 | 0.021 | 2.000 | 37.628 | 29.841 | 0.080 | 0.021 | 10241.505 | 1945.481 | 1.000 | |
| 4 | 84.006 | 9.998 | 3.358 | 0.644 | 2.000 | 37.427 | 30.397 | 0.071 | 0.021 | 9937.900 | 1872.277 | 1.000 | |
| 5 | 95.833 | 0.021 | 1.536 | 0.607 | 2.000 | 37.991 | 30.415 | 0.017 | 0.020 | 7485.236 | 1949.050 | 1.000 | |

**Note:** PGPR was maintained at a constant level of 2.00% in all formulations. The slight deviation from 100% in the component sum is attributable to rounding.
